# A New Selection Strategy to Enrich Dopaminergic Neurons Facilitates Comparisons Between Patient iPSC-Derived Lines

**DOI:** 10.3390/ijms27219394

**Published:** 2026-10-22

**Authors:** Ellen Hertz, Gani Perez, Krystyna Rytel, Martha Kirby, Stacie Anderson, Stephen Wincovitch, Tim Ahfeldt, Joel Blanchard, Ying Hao, Ziyi Li, Kate Andersh, Yue Andy Qi, Ellen Sidransky, Yu Chen

**Affiliations:** 1 Medical Genetics Branch, National Human Genome Research Institute, National Institutes of Health, Bethesda, MD 20892, USA; ellen.hertz@outlook.com (E.H.); idp2026@nyulangone.org (G.P.); knr2126@cumc.columbia.edu (K.R.); 2 Aligning Science Across Parkinson’s (ASAP) Collaborative Research Network, Chevy Chase, MD 20815, USA; ahfeldt@gmail.com (T.A.); joel.blanchard@mssm.edu (J.B.); 3 Core Facilities, National Human Genome Research Institute, National Institutes of Health, Bethesda, MD 20892, USA; makirby@mail.nih.gov (M.K.); staciea@mail.nih.gov (S.A.); wincovis@mail.nih.gov (S.W.); 4 Nash Family Department of Neuroscience at Mount Sinai, New York, NY 10029, USA; 5 Center for Alzheimer’s and Related Dementias (CARD), National Institute on Aging and National Institute of Neurological Disorders and Stroke, National Institutes of Health, Bethesda, MD 20892, USA; ying.hao@nih.gov (Y.H.); ziyi.li@nih.gov (Z.L.); kate.andersh@nih.gov (K.A.); andy.qi@nih.gov (Y.A.Q.); 6 DataTecnica LLC., Washington, DC 20037, USA

**Keywords:** iPSC, dopaminergic neuron differentiation, *GBA1*, Parkinson disease, neomycin resistance, Gaucher disease

## Abstract

Comparative studies of dopaminergic (DA) neurons differentiated from induced pluripotent stem cells (iPSCs) derived from patients discordant for parkinsonism, especially siblings, provide a valuable model to explore factors underlying Parkinson disease (PD) pathogenesis. However, such studies are complicated by the inherent heterogeneity in differentiation efficiency among iPSC lines derived from different individuals. To address this technical challenge, we devised a selection strategy to enrich DA neurons expressing tyrosine hydroxylase (TH). A neomycin resistance gene (*neo*) was inserted at the C-terminus of the *TH* gene, placing its expression under the endogenous promoter. Using iPSC-derived neurons from a healthy individual (HT711), we achieved selective enrichment of TH+ neurons through geneticin selection without compromising differentiation capacity, as demonstrated by Western blots, immunocytochemistry and flow cytometry. We then applied the method to iPSCs derived from two siblings of HT711, one with Gaucher disease (GD) alone and one with both PD and GD. Quantification of multiple DA neuron markers through whole-cell proteomics confirmed their comparable enrichment in the three lines, demonstrating that the new method can be applied to iPSC lines from different individuals to standardize DA neuron cultures. While we focus on methodological developments here, a companion analysis of the same whole-cell proteomics dataset, examining molecular features potentially associated with disease statuses, has been reported separately.

## 1. Introduction

Biologically relevant in vitro models of dopaminergic (DA) neurons are of significant scientific interest due to the pivotal role of these cells in common diseases like Parkinson disease (PD). While the progressive degeneration of DA neurons in the substantia nigra is a defining feature and cause of the cardinal motor symptoms in PD [1,2], the cellular mechanisms responsible for this neuronal loss remain largely elusive, although genetic vulnerability plays a role [3]. Comparative studies of DA neurons differentiated from induced pluripotent stem cells (iPSCs) derived from individuals discordant for PD offer an opportunity to study underlying pathogenic mechanisms in the affected cell type, while preserving a permissive or resistant genetic background [4]. However, despite progress in differentiation methods, inherent heterogeneity in DA neuron differentiation efficiency among iPSC lines and culture batches complicates the interpretation and reproducibility of results [5,6]. This variability may be related to donors’ overall genetic background rather than the genotypes of research interest, and this phenomenon is also observed in studies of other iPSC-derived cell lineages [7]. Efforts have been focused on refining differentiation protocols with timely addition of signaling molecules mimicking in vivo embryonic development [6,8], but critical events for dopaminergic patterning, such as the level of *Wnt* activation [9,10,11], seem to be an inherent cell line characteristic [12]. Furthermore, the final fraction of DA neurons is often limited in the culture and is mixed with proliferating neural progenitor cells as well as other types of cells. A single-cell RNA-sequencing analysis of iPSC-derived DA neuron cultures from 95 patient-derived iPSC lines revealed substantial line-to-line variation, with an average of 80% of differentiated cells being MAP2-positive, and only 20% also expressing TH [13]. Consequently, disease-related changes in DA neurons may sometimes be obscured by culture heterogeneity, and the inherent differentiation differences between iPSC lines call for methods to standardize DA neuron culture for comparative studies.

Various methods have been employed to overcome this heterogeneity. Optimization of differentiation conditions for each cell line has been suggested, but this is time-consuming and introduces potential biases [14]. Expression of lineage-determining transcription factors has been successful in generating glutamatergic-like neurons [15], but similar protocols for inducing DA neurons requires combinations of transcription factors together with exogenous patterning cues [16,17,18]. The DNA cross-linker mitomycin-C (MMC) has been used to remove proliferating progenitor cells in differentiating DA neuron cultures [19,20]. Fluorescence activated cell sorting (FACS) has been used for purification of DA progenitor cells for clinical purposes [21,22] or DA neurons expressing tyrosine hydroxylase (TH) [23] and those genetically engineered to express fluorescent proteins concomitant with TH [24,25]. In addition, a neomycin (*neo*) resistance gene driven by a *MAP2*-promoter has previously been inserted to the safe-harbor AAVS1 site in iPSCs and used for selection of DA neurons [26], despite MAP2 being a pan-neuronal marker [13].

In this project, we developed a geneticin-based selection strategy to enrich DA neurons expressing tyrosine hydroxylase (TH) and evaluated it using iPSC lines from three sisters. One sister is a healthy control, and two sisters had Gaucher disease caused by biallelic mutations in *GBA1* which is the most common genetic risk factor for PD. One of the sisters also developed PD. To increase the purity of iPSC-differentiated DA neurons from the sisters, we inserted a neomycin resistance gene (*neo*) into the *TH* locus using CRISPR/Cas9-based genetic engineering, placing its expression under the control of the endogenous *TH* promoter. The *neo* insertion had no impact on iPSC differentiation into TH+ DA neurons, and the expression of aminoglycoside 3′-phosphotransferase II (APT II), encoded by *neo*, was concurrent with TH. Successful DA neuron enrichment with geneticin selection was confirmed in cultures using immunocytochemistry, Western blots, flow cytometry and whole-cell proteomics. While this paper describes the development and implementation of the neomycin selection approach for DA neuron enrichment and analyzed the whole-cell proteomics data to demonstrate increased levels of multiple DA neuron markers after geneticin selection, a companion analysis of the whole-cell proteomics data, investigating molecular features potentially associated with the different diseases, has been reported in a prior publication focusing on disease mechanisms [27].

## 2. Results

### 2.1. Clinical Samples

**Figure 1:**
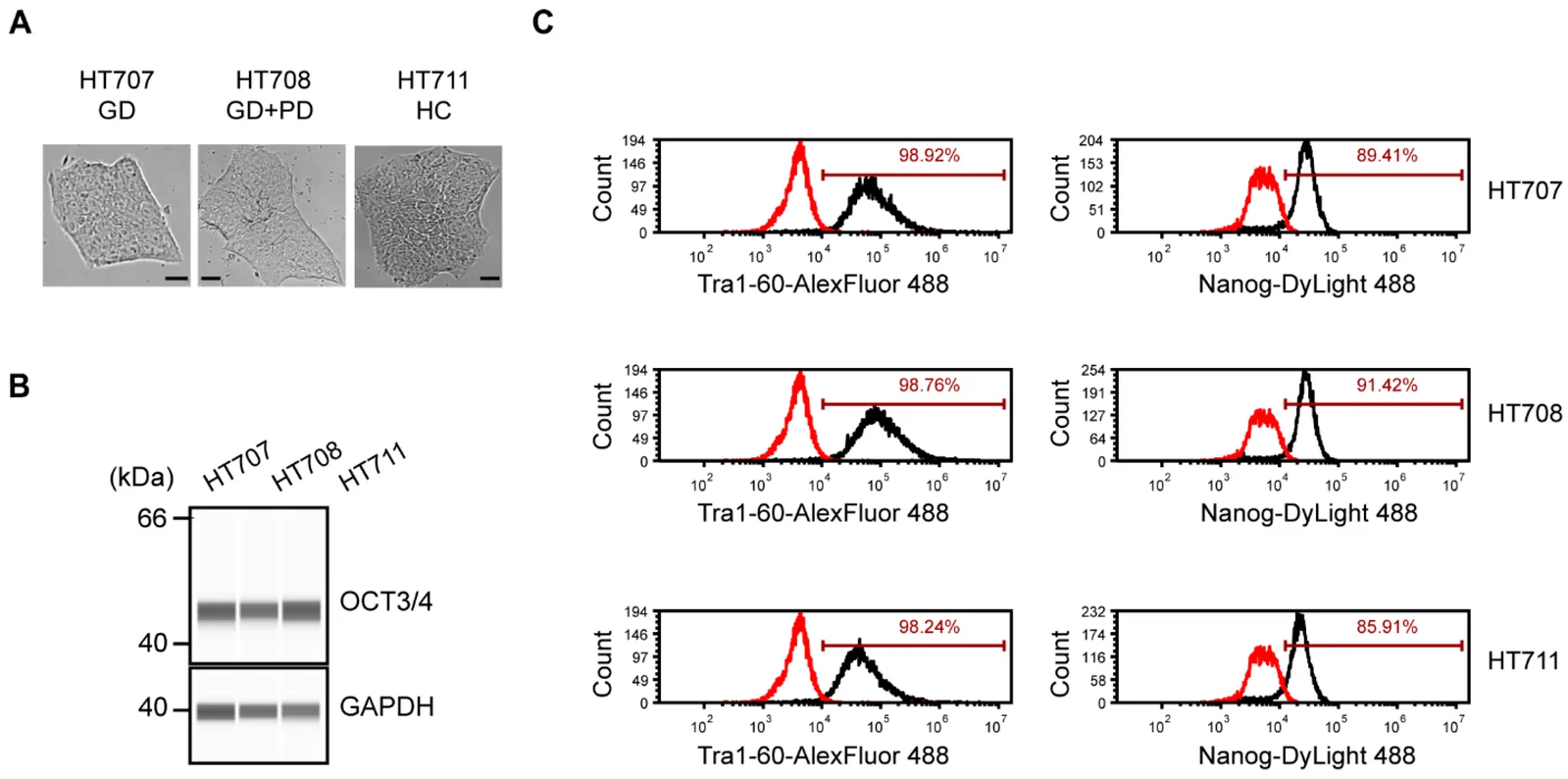
Characterization of patient-derived iPSC lines. (**A**) Brightfield images of iPSCs (scale bars, 10 µm). (**B**) The expression of pluripotency marker OCT3/4 in all iPSC lines demonstrated by Western blotting. (**C**) Quantification of the expression of pluripotency markers Tra1-60 and Nanog in all iPSC lines with flow cytometry. Black peaks represent staining intensities with indicated antibodies, and red peaks represent isotype controls.

Skin fibroblasts were obtained from the sister with GD only (HT707) at age 60, the sister with GD + PD (HT708) at 56 (after PD diagnosis), and the healthy control sister (HT711) at 58 (Supplementary Table S1). Reprogramed iPSCs showed expected morphology (Figure 1A) with concordant expression of the pluripotency markers OCT3/4, Tra1-60 and Nanog (Figure 1B,C).

### 2.2. Genetic Engineering of iPSC Lines to Enable DA Neuron Enrichment Through Geneticin Selection

**Figure 2:**
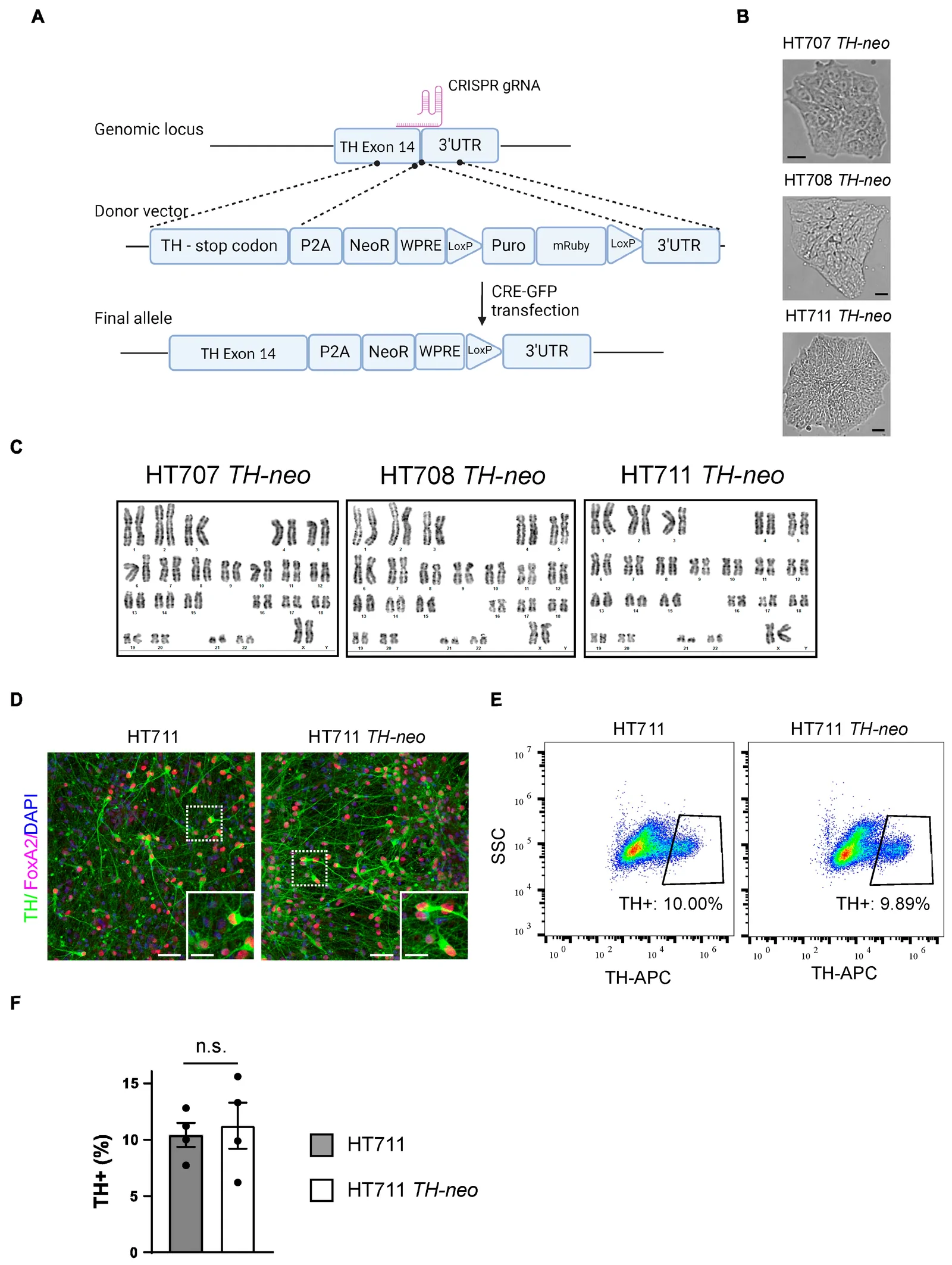
Generation and characterization of *TH-neo*-edited iPSC lines. (**A**) CRISPR-mediated knock-in of *neo* in the TH locus. (**B**) Morphology of *TH-neo* iPSC lines (scale bar, 10 µm). (**C**) Normal karyotypes of *TH-neo* iPSC lines. (**D**) Immunocytochemistry (ICC) of TH+ cells in HT711 and HT711 *TH-neo* on day 37 of differentiation (scale bar, 50 µm; inset scale bar, 25 µm). (**E**,**F**) Quantification of TH+ cells in HT711 and HT711 *TH-neo* on day 37 of differentiation (*t*-test, *n* = 4 independent differentiations).

To address the inherent variability in DA neuron differentiation among iPSC lines reprogrammed from different donors, we developed a method to enrich *TH*-expressing (TH+) DA neurons within iPSC-derived DA neuronal cultures. Employing CRISPR-Cas9 and homology-directed repair (HDR), we inserted a *neo* gene into the *TH* locus after exon 14, following a P2A self-cleavage peptide. Anticipating the expression of *neo*, driven by the endogenous *TH* promoter, to be restricted to TH+ DA neurons, we aimed to facilitate their enrichment with geneticin selection (Figure 2A). Because the *TH* gene is transcriptionally silent in iPSCs, our HDR donor vector included an EF1α promoter-driven selection cassette. This cassette included a puromycin resistance gene and a red fluorescent protein mRuby, separated by a T2A self-cleavage peptide. This design aimed to aid the identification of correctly edited iPSCs with puromycin selection and FACS identification of mRuby+ iPSCs. Furthermore, the selection cassette was flanked by loxP sites to assist its removal after the edited iPSCs were identified. After the co-transfection of the HDR donor vector and the CRISPR plasmids encoding Cas9 protein and transcribing sgRNAs, iPSCs with HDR donor vector integration were enriched with puromycin selection. Enriched iPSCs were then transfected with a plasmid encoding GFP-tagged CRE-recombinase to remove the selection cassette. Individual iPSCs expressing CRE-GFP were sorted into 96-well plates via FACS to derive single cell clones. Edited iPSC clones were further analyzed using PCR to confirm the intended insertion. The edited iPSC clonal lines were referred to as HT707 *TH-neo*, HT708 *TH-neo*, and HT711 *TH-neo*, respectively. Their morphology remained unchanged, and they exhibited normal karyotypes (Figure 2B,C).

### 2.3. Integration of the Neo Gene Does Not Interfere with Differentiation of iPSCs into DA Neurons

To evaluate the potential influence of *neo* gene integration on iPSC differentiation into DA neurons, HT711 and HT711 *TH-neo* were differentiated into DA neurons following a protocol developed by Kim et al. [9]. The differentiation efficiency, measured by the percentage of TH+ DA neurons on day 37 of differentiation, was comparable between HT711 and HT711 *TH-neo*, as evaluated by immunofluorescence and flow cytometry. This indicates that *neo* gene integration did not perturb differentiation capacity (Figure 2D–F).

### 2.4. Concurrent Expression of Tyrosine Hydroxylase and Neo in TH-Positive DA Neurons

**Figure 3:**
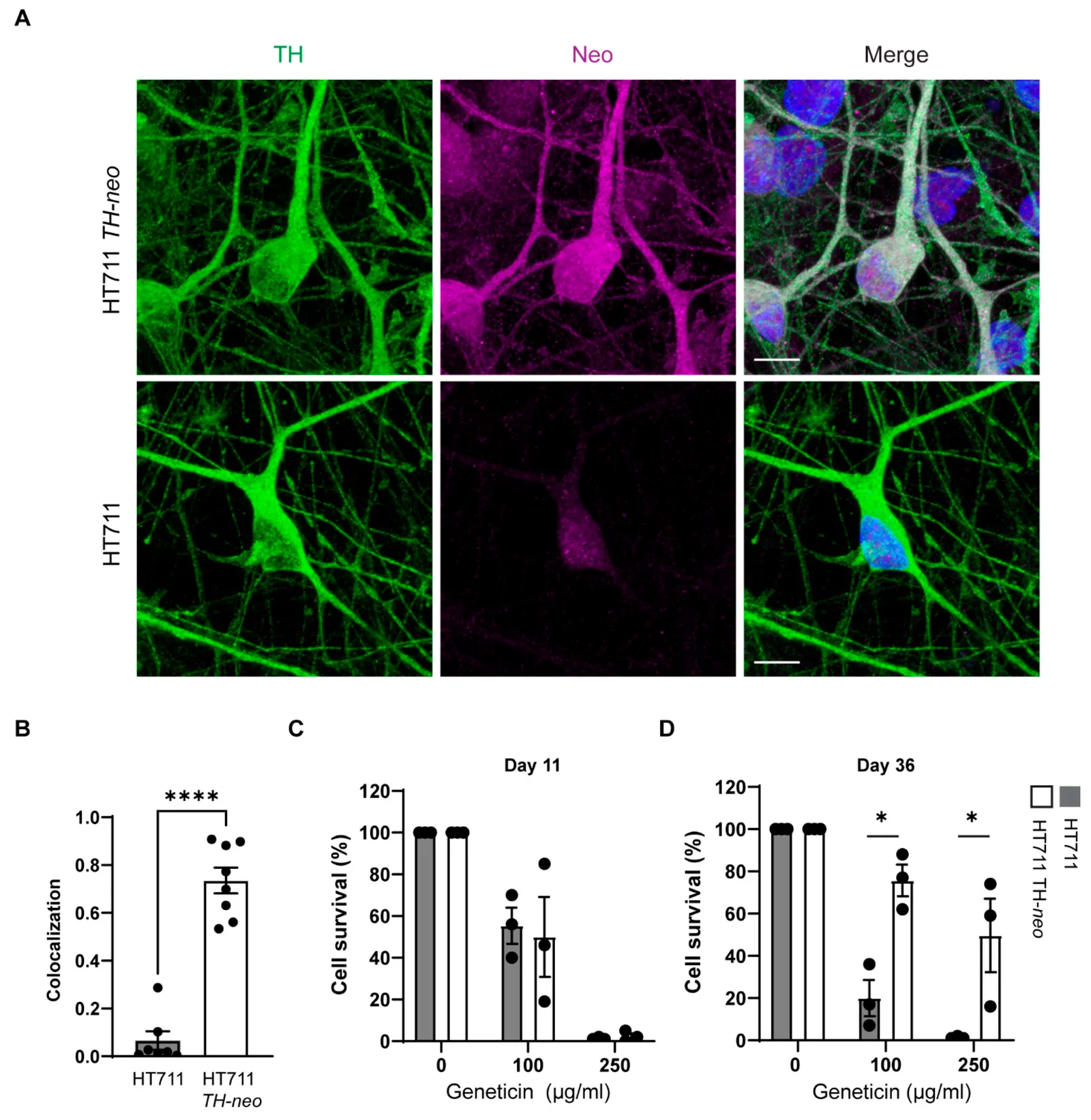
*Neo* expression is limited to TH+ neurons. (**A**) The expression of *neo* is limited in TH+ neurons as demonstrated with ICC (scale bars, 10 µm). (**B**) Quantification of colocalization. No correlation is detected in HT711 (*t*-test, ****, *p* < 0.001, *n* = 3 independent differentiations). (**C**) Geneticin eliminates both HT711 and HT711 *TH-neo* neural progenitor cells (*n* = 3 independent differentiations). (**D**) Dose-dependent survival of HT711 *TH-neo* DA neurons following geneticin treatment (ANOVA, * *p* < 0.05, *n* = 3 independent differentiations).

For efficient enrichment of DA neurons through geneticin selection, it is crucial for the *neo* gene to be expressed exclusively in TH+ DA neurons. Immunostaining on day 37 of differentiation revealed precise expression of aminoglycoside 3′-phosphotransferase II (APT II), the protein encoded by *neo*, exclusively in TH+ DA neurons differentiated from HT711 *TH-neo*. This indicates the expression of APT II was under the control of the TH promoter (Figure 3A,B). In contrast, TH+ DA neurons from HT711 showed minimal background staining, confirming the specificity of the APT II antibody. To validate geneticin selection stress on proliferating neuronal precursors and neurons lacking TH and APT II, we evaluated cell viability at both the neuronal precursor and neuronal stages using the CellTiter-Glo luminescent cell viability assay. At the neuronal precursor stage (day 16), prior to the onset of TH expression, geneticin treatment similarly reduced the viability of HT711 and HT711 *TH-neo* neuronal precursors (Figure 3C). At a concentration of 250 μg/mL, geneticin effectively eliminated neuronal precursors, demonstrating its efficacy in removing proliferating precursors from DA neuron cultures. When assessed on day 36 of neuronal differentiation, after 10 days of treatment with 250 μg/mL of geneticin, ~50% of the cells in HT711 *TH-neo* DA neuron cultures survived, whereas nearly all cells in HT711 DA neuron cultures perished, indicating successful elimination of neurons not expressing APT II through geneticin selection (Figure 3D).

### 2.5. Geneticin Treatment Increases the Fraction of TH+ DA Neurons

**Figure 4:**
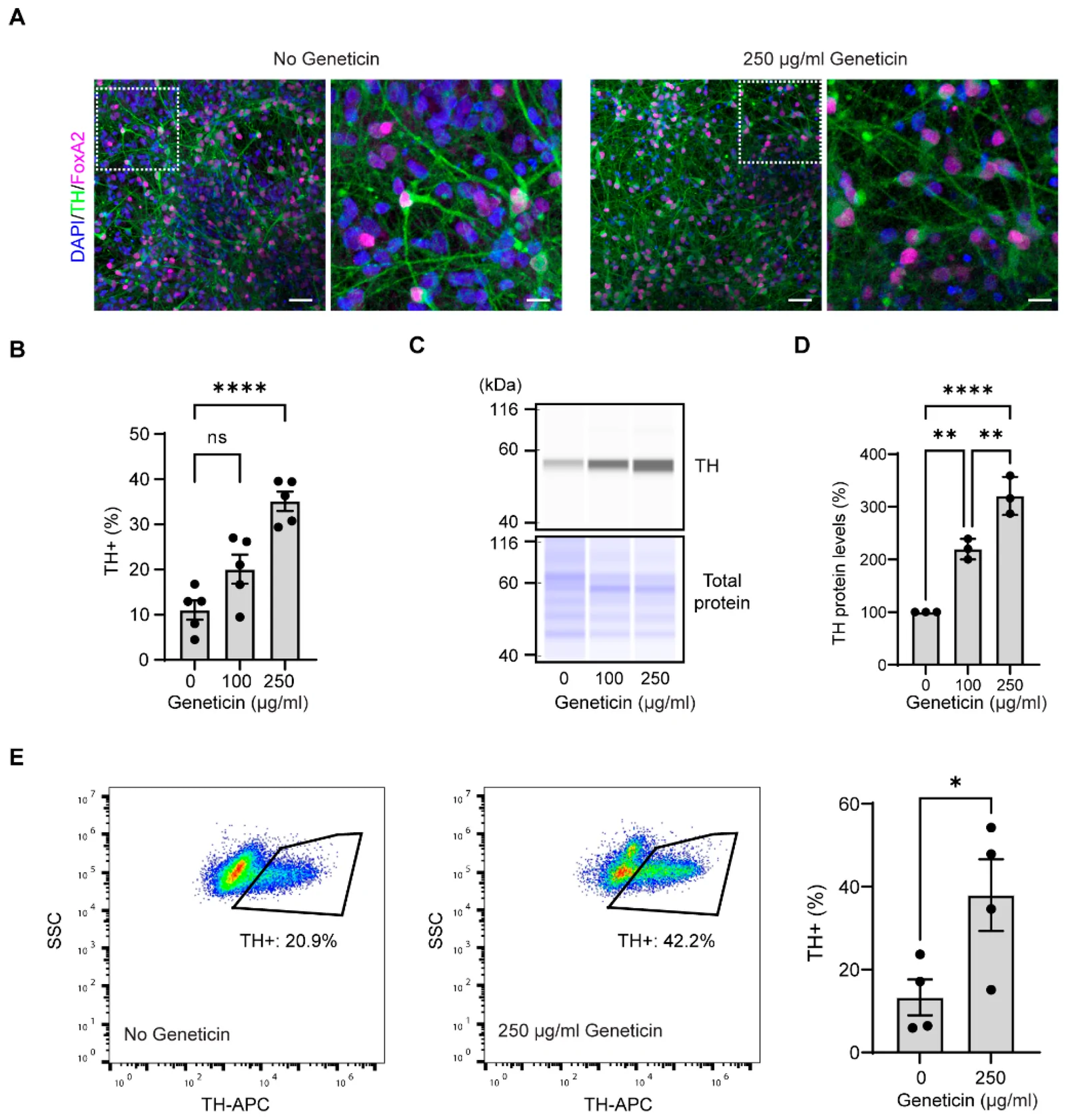
Enrichment of TH+ neurons with geneticin treatment. (**A**) Confocal image of TH+ DA neurons from the HT711 TH-neo line with and without geneticin treatment. Note the reduction in nuclei without accompanying TH staining (scale bar, 45 µm; inset scale bar, 15 µm). (**B**) Quantification of TH+ DA neurons in ICC (1-way ANOVA, **** *p* < 0.001, *n* = 5 fields of view from 2 independent differentiations). (**C**,**D**) Western blot analysis of TH expression. Data is normalized to cells without geneticin treatment (1-way ANOVA, **** *p* < 0.0001, ** *p* < 0.01, *n* = 3 independent differentiations). (**E**) Flow analysis of TH+ cells. (*t*-test, * *p* < 0.05, *n* = 4 independent differentiations.).

To illustrate the enrichment of TH+ DA neurons after geneticin selection, we quantified the percentage of TH+ DA neurons using both immunofluorescence and flow cytometry analyses. Immunofluorescence studies revealed that treatment with 250 μg/mL of geneticin for 10 days increased the fraction of TH+ cells from 10% to 35% (Figure 4A,B), while flow cytometry demonstrated an increase from 10% to 45% (Figure 4E) on day 37. Furthermore, Western blot analysis showed a dose-dependent increase in the expression of TH following geneticin selection, with an approximately 2.5-fold increase observed at a concentration of 250 μg/mL (Figure 4C,D).

### 2.6. Standardization of DA Neurons Across Patient-Derived iPSC Lines Was Confirmed by Cellular Proteomics

To assess the cellular composition in DA neuron culture pre- and post-geneticin selection, we employed a robust proteomics pipeline involving automated sample preparation and data-independent acquisition (DIA) coupled with a high-field asymmetric waveform ion mobility spectrometer (FAIMS) interface, and an optimized library-free DIA database search strategy [28]. We treated the cultures with geneticin or vehicle for 10 days and continued to mature them to day 75 for cellular proteomics analysis. The pipeline detected over 8800 proteins, indicating a comprehensive coverage of the cellular proteome.

**Figure 5:**
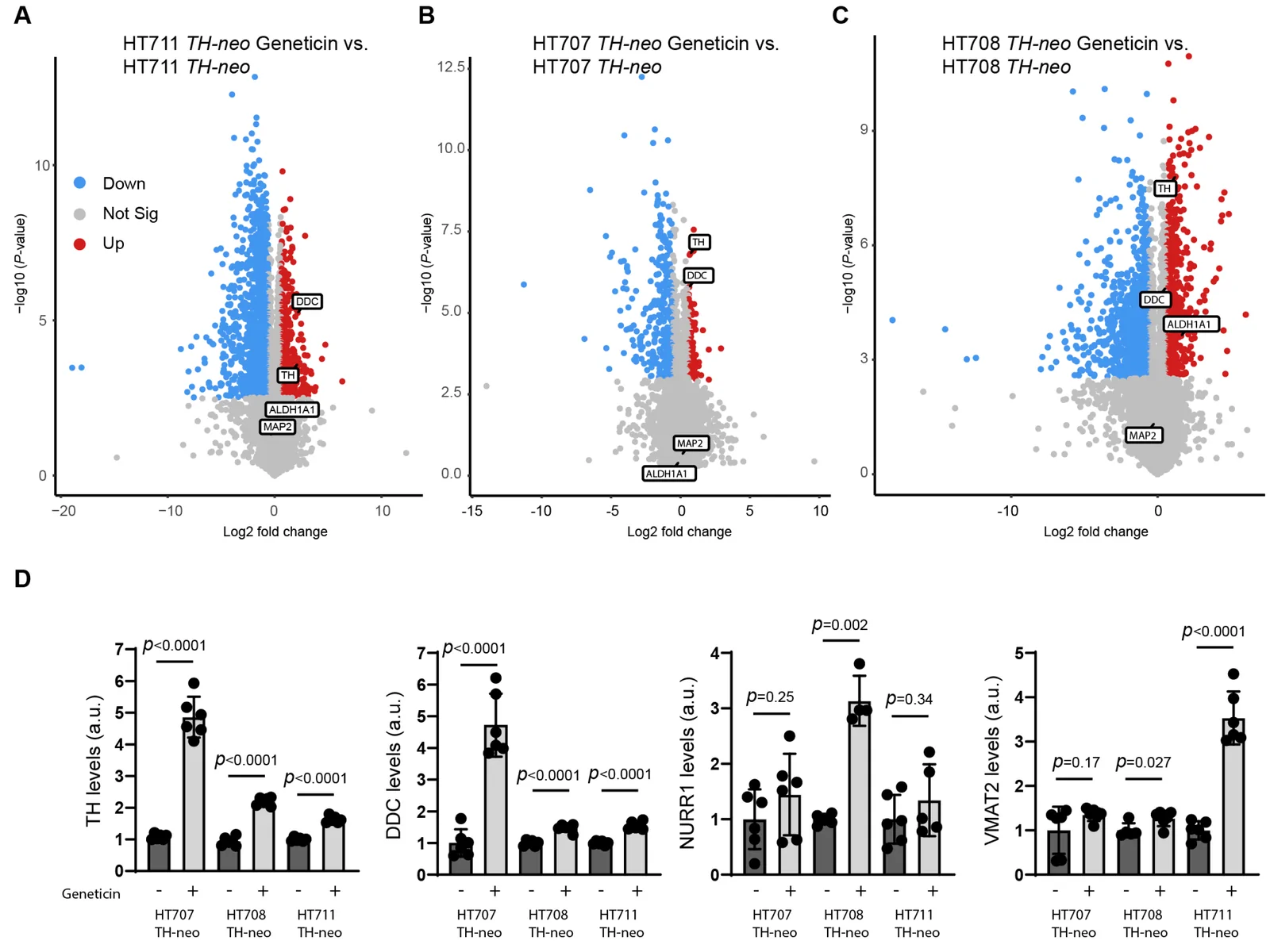
Enrichment of dopaminergic markers via geneticin selection. (**A**–**C**) Volcano plots showing enrichment of dopaminergic markers after treatment with geneticin for HT711 *TH-neo*, HT707 *TH-neo,* and HT708 *TH-neo*. (**D**) Bar graphs showing TH, DDC, NURR1, and VMAT2 levels before and after DA neuron enrichment with geneticin (mean ± SD, Welch’s *t* test).

After geneticin treatment, all three cell lines exhibited increased abundance of TH (711 *TH-neo* log2FC 2.14, adj. *p* < 1 × 10^−3^; 707 *TH-neo* log2FC 0.70, adj. *p* < 2.5 × 10^−5^; 708 *TH-neo* log2FC 1.22, adj. *p* < 3 × 10^−6^) and dopa decarboxylase (DDC), which coverts L-DOPA to dopamine (711-*TH-neo* log2FC 2.14, adj. *p* < 8 × 10^−5^; 707-*TH-neo* log2FC 0.68, adj. *p* < 1 × 10^−4^; 708-*TH-neo* log2FC 1.75, adj. *p* < 5 × 10^−7^) (Figure 5A–D). Additionally, enhanced expressions of other DA neuronal markers were also evident, including ALDH1A1—a specific marker for PD-vulnerable A9 DA neurons—in two of the lines (708-*TH-neo* log2FC 1.60, adj. *p* < 0.002; 711-*TH-neo* log2FC 2.32, adj. *p* = 0.013), as well as Nurr1, and VMAT2 (Figure 5D). In contrast, the pan-neuronal markers MAP2 and TUBB3 were not significantly enriched. This is consistent with a previous report showing that a large proportion of iPSC-derived neurons were MAP2-positive but lacked TH expression [13].

## 3. Discussion

Thorough assessments of rare or unique clinical cases offer valuable insight into underlying molecular mechanisms [29,30]. Disease-relevant cell types differentiated from different patient-derived iPSC lines can serve as models that recapitulate individual-specific molecular signatures. To facilitate accurate downstream comparisons between individuals, we addressed the substantial differentiation heterogeneity inherent in iPSC lines, even among lines derived from genetically related individuals. Previous advancements, including optimized differentiation protocols for specific iPSC lines from healthy controls, deep phenotyping of selected cell lines with the desired characteristics [31], and data atlases generated from numerous iPSCs in large-scale collaborative projects [13], may not be applicable when studying rare diseases. Consequently, we aimed to develop a tool capable of enriching the desired cell type from any iPSC line, thereby reducing expected heterogeneity between patient-derived samples. After placing the expression of *neo* under the control of the TH promoter, we first verified that *neo* did not interfere with differentiation capacity on day 37. Whole-cell proteomics on day 75 also indicated close clustering of HT711 and HT711-TH-neo, suggesting differentiation capacity remained largely unchanged [27]. We then confirmed the exclusive expression of *neo* in TH+ DA neurons. The effectiveness of geneticin selection was then demonstrated by comparing cell survival between the parental line and the corresponding *TH-neo* line. While the initial fraction of TH+ neurons in HT711 was lower than reported by Kim et al., it is comparable to levels reported in larger sets of iPSC-derived neurons [13]. Optimization of CHIR levels for each iPSC line has been proposed to maximize levels of DA neurons. However, when comparing different iPSC lines, this is not feasible, prompting us to develop a method more applicable to patient-derived neurons. We showed enrichment of TH+ neurons using multiple methods. Immunostaining confirmed an increased fraction of TH+ neurons post-geneticin selection, due to a decrease in DAPI-stained nuclei lacking TH. Flow cytometry analysis also revealed a comparable degree of enhancement. Compared to immunostaining, flow cytometry analysis required similar hands-on time, but analyzed a much larger number of neurons. Cell viability assay showed ~50% survival of HT711-*TH*-neo neurons and near-total death in HT711 neurons after selection. These data indicate that geneticin selection enriches TH+ neurons by eliminating TH- ones, and the reduced yield is a worthwhile trade-off for the gain in purity. The elimination of non-edited HT711 also indicates that, although not all cells were positive in immunostaining FACS after selection, this is likely due to limited sensitivity of the staining. In addition, the donor vector does not include a promotor and hence *neo* is only expressed through the endogenous TH promotor. Because geneticin selection has been shown not to alter the electrophysiological properties of TH+ neurons differentiated from an iPSC line with a neomycin resistance gene driven by the human MAP2 promoter inserted at AAVS1 [26], we believe our method offers a valuable tool for studies comparing TH+ neurons differentiated from multiple patient iPSC lines.

This new method, complimentary to previous approaches such as coupling a fluorescent reporter to TH expression or surface marker-based FACS [21], offers the advantage of cell enrichment without the need for dissociation into single cells, and can also be applied at any point of maturation after the onset of TH expression. This provides the opportunity for drug screening in cell cultures with intact synapses. A comparison of enrichment methods can be found in Table 1.

This new method has successfully facilitated a variety of multiomic analyses focused on GD and PD phenotypes, as reported earlier by our group [27], demonstrating its utility for standardizing DA neurons cultures across iPSC lines from multiple individuals. The current report focuses entirely on the development and validation of the enrichment methodology itself—specifically, the engineering of iPSC lines with a neomycin resistance cassette under the TH promoter, and the characterization of the resulting enriched dopaminergic (DA) neuron populations. These methodological developments provide a valuable tool for the broader scientific community engaged in disease modeling using iPSC lines derived from multiple individuals.

## 4. Materials and Methods

All materials used together with the vendors and catalog numbers are summarized in Supplementary Table S5. Original data and protocol links can be found in https://zenodo.org/records/18876867 (accessed on 9 October 2026).

### 4.1. Obtaining and Genotyping Fibroblasts and Reprogramming into iPSCs

The three participants were evaluated under the IRB-approved clinical protocol 86HG0096 at the National Institutes of Health, and each provided informed consent prior to participation. Sanger sequencing of the glucocerebrosidase gene was performed as previously described.

Fibroblasts were obtained by punch biopsy and cultured in Dulbecco’s Modified Eagle’s Medium (DMEM) supplemented with 15% fetal bovine serum (FBS) and with 1× penicillin–streptomycin at 37 °C. The CytoTune™-iPS 2.0 Reprogramming System, based on non-integrative Sendai virus, was used to generate iPSCs at the National Heart, Lung and Blood Institute’s iPSC Core according to standard procedures.

### 4.2. Genomic Engineering of iPSCs

iPSCs were maintained in Essential 8 Medium on Vitronectin-coated plates without addition of antibiotics. Media was exchanged daily, and cells were kept at 37 °C and 5% CO_2_. Cells were passaged by dissociation in 0.5 mM EDTA every 3–4 days and resuspended in Essential 8 Medium supplemented with Y-27632 dihydrochloride. For CRISPR-Cas9 editing, iPSCs were dissociated to single cells with StemPro-Accutase and seeded in Essential 8 Medium with Y-27632 at a density of 200,000 cells/cm^2^ two hours prior to transfection with Lipofectamine Stem Transfection Reagent. A donor plasmid (RRID: Addgene_247669) was generated, similar to that previously described by Ahfeldt et al. [32], with a 5′ TH homology arm, a 2A self-cleaving peptide sequence followed by *neo*-cassette, and a WPRE sequence. In addition, a floxed selection cassette including a puromycin resistance cassette and mRuby sequence, and a 3′ TH homology arm (Figure 1A), was co-transfected with vectors expressing gRNA sequences (RRID: Addgene_247673 and Addgene_247674) targeting the second to last codon of the human TH-locus (excluding the stop codon) and Cas9. Off-target predictions can be found in Supplementary Table S2. Cells were replated the next day in StemFlex, supplemented with Y-27632, and 24 h later, selection with 0.25 μg/mL puromycin was initiated. Puromycin-resistant clones were allowed to replicate until near-confluence and then pooled. CRE-mediated excision of the selection cassette was performed as described above, and single clones were collected by FACS on a Sony SH800 cell sorter (Sony, Tokyo, Japan) the following day. Gates were determined by non-transfected control iPSCs. Single clones were sorted into a laminin-521-coated plate with Stemflex supplemented with 10 μM Y-27632, 5 μM Emricasan, 1× penicillin–streptomycin, 0.7 μM ISRIB and 1× polyamines and incubated for 3 days. Then, half of the media was changed with Stemflex supplemented with penicillin–streptomycin, ISRIB and polyamines. After this, the media was changed every three days with Stemflex alone, until the surviving clones reached at least 50% confluency and could be expanded.

Clones were screened for the correct knock-in by PCR, amplifying both cleavage sites. DNA was extracted from flash-frozen pellets using 30 μL of QuickExtract and samples were heated to 60 °C for 15 min, 65 °C for 15 min and 99 °C for 10 min. AmpliTaq Gold 360 Master Mix (ThermoFisher Scientific, Waltham, MA, USA 4398881) was mixed 1:2 with H_2_O, 0.25 μM of forward and reverse primers and 1 μL of cell extract. The primer sequences and PCR cycle conditions can be found in Supplementary Table S3.

### 4.3. Karyotyping

iPSC clones, which were confirmed for knock-in of the construct, were dissociated and plated as described above at 1 × 10^6^ cells/well in 6-well plates in Stemflex with 10 μM Y-27632. The following afternoon, Colcemid (1:100) was added, and cells were karyotyped using standard procedures at the National Human Genome Research Institute Cytogenetics Core Facility.

### 4.4. Directed Differentiation into Midbrain Dopamine Neurons (mDA)

The differentiation protocol for dopaminergic neurons was adopted from Kim et al. with minor adaptations [9]. In short, iPSCs were dissociated into single cells with Accutase and plated on Geltrex-coated plates at 400,000 cells/cm^2^ in Neurobasal Medium with 1× GlutaMAX, 1× N2, 1× B-27 without Vitamin A supplemented with 500 ng/mL of human Sonic Hedgehog/Shh (C24II) N-Terminus, 250 nM LDN 193189 dihydrochloride, 2 μM A 83-01, 0.7 μM CHIR99021, 2 μM SAG 21k, 10 μM Y-27632 and 5 μM Emricasan. On day 4, CHIR99021 was increased to 3 μM. On days 8 and 9, cells were cultured in Neurobasal/GlutaMAX/1X N2/B-27 with only 3 μM of CHIR99021. On day 10, media was changed to Neurobasal/GlutaMAX/B-27 with 20 ng/mL of brain-derived neurotrophic factor (BDNF), 20 ng/mL of glial cell line-derived neurotrophic factor (GDNF), 200 μM ascorbic acid, 1 ng/mL of transforming growth factor type β3 (TGFβ3), 200 μM dibutyryl cAMP and 3 μM CHIR99021. On day 11, cells were sub-plated at 800,000 cells/cm^2^ with Accutase in the same media with added Y-27632 and Emricasan. On day 13, CHIR99021 was substituted with 10 μM DAPT. Cells were dissociated for the last time on day 25 and seeded at 200,000 cells/cm^2^. On day 27, selection with geneticin/GENETICIN was initiated and kept for 10 days until experiments were conducted on day 37, unless otherwise specified. For experiments conducted on day 75 of differentiation, media was changed once per week with the addition of 1 μM 5-fluoro-2′-deoxyuridine and 1 μM uridine to prevent division of non-differentiated cells.

### 4.5. Cell Viability Assay—Cell-Glo

Cells were seeded in white opaque 96-wells with clear bottoms after regular dissociation on day 11 at 800,000 cells/cm^2^ and day 25 at 200,000 cells/cm^2^, respectively, and treated with geneticin. On day 19 or 36, CellTiter-Glo was resuspended with buffer, added 1:1 vol/vol directly to the media, and placed on an orbital shaker for 2 min. After 10 min of incubation at RT in the dark, luminescence was detected on Flexstation 3 (Molecular Devices, San Jose, CA, USA).

### 4.6. Western Blots—Automated System

Western blots were processed using an automated system as follows. Dopaminergic neurons and iPSC pellets were resuspended in Triton-X (1% Triton X-100, 10% glycerol, 150 mM NaCl, 25 mM HEPES pH 7.4, 1 mM EDTA, 1.5 mM MgCl_2_), supplemented with one EDTA-free protease inhibitor tablet per 10 mL of buffer or RIPA buffer, lysed through thorough pipetting or 3 freeze–thaw cycles and centrifuged at 21,000× *g* for 15 min at 4 °C. The supernatant was transferred to new tubes. Protein concentration was determined by BCA according to the manufacturer’s instructions and absorbance values were detected on the FlexStation^®^ 3 Multi-Mode Microplate Reader (Molecular Devices).

Comprehensive instructions for Jess are found with the kit and were run according to instructions in chemiluminescent RePlex mode with EZ Standard Pack 1. In brief, the DTT was prepared with 40 μL of deionized water and the biotinylated ladder with 20 μL of deionized water respectively. Fluorescent 5X Master Mix was prepared with 20 μL of Sample Buffer and 20 μL of the DTT solution. Samples were prepared by combining one-part fluorescent master mix with four parts of the sample to a final concentration of 1 μg/μL. Samples and a biotinylated ladder were incubated for 5 min at 95 °C. Fluorescence Separation Capillary Cartridges (12–230 kDa) were loaded according to instructions in the RePlex pack with 3 μL of sample per well. Primary antibodies (details in Supplementary Table S4) were diluted in antibody diluent 2 and normalization was based on total protein or reference gene. Plates were centrifuged for 5 min at 1000× *g* before addition of wash buffer, luminol-peroxide mix and RePlex reagent. Visualization was performed with SW Compass (Version 6.3.0, Simple Western, San Jose, CA, USA). Original files can be found in Supplementary Figure S1.

### 4.7. Flow Cytometry

Neurons were washed in PBS and incubated with Membrite prestain solution, diluted 1:1000 in HBSS, for 5 min at 37 °C. Prestain solution was changed to staining solution and incubated for another 5 min at 37 °C. Cells were dissociated with prewarmed papain with DNAse, Y-27632 and Emricasan for 10 min at 37 °C. The reaction was quenched in DMEM/Glutamax with 10% fetal bovine serum and collected by centrifugation at 400× *g* for 5 min. The pellet was resuspended in 4% paraformaldehyde and incubated for 10 min at RT on a rolling wheel. Cells were resuspended in DMEM with 10% FBS and spun at 500× *g* for 5 min after which the cells were incubated in permeabilization buffer (1% BSA, 0.1% saponin in PBS, 10% normal donkey serum) for 30 min at RT and thereafter labeled with tyrosine hydroxylase antibody or isotype controls (1:400) overnight at 4 °C. Cells were washed twice with 2% BSA and 1.5 mM EDTA in PBS and then incubated with diluted Alexa647-conjugated secondary antibody, for 1 h at RT. Finally, cells were washed as described above with NucBlue Fixed Cell ReadyProbes Reagent (DAPI) in the second wash and resuspended in wash buffer for flow cytometry analysis on a Beckman Coulter Cytoflex-LX. Initial gating included Membrite-positive cells, as a secondary gate DAPI positivity was used in order to exclude duplicates. Finally, the cells were analyzed for the fluorescent intensity of TH using Flow-Jo v10 (RRID:SCR_008520) with the isotype control intensity used to define positive vs. negative cells.

### 4.8. Immunocytochemistry (ICC)

Cells were seeded in Geltrex-coated 8-well imaging chambers on differentiation day 25. After selection as described above, cells were fixed in 4% paraformaldehyde (15 min at RT) and washed for 3 × 5 min in PBS on day 37. Thereafter, cells were blocked for 1 h at RT and incubated overnight with primary antibodies diluted in blocking buffer (1% BSA, 0.1% saponin in PBS) at 4 °C. The primary antibodies used were tyrosine hydroxylase (1:400), FoxA2 or APT II. The following day, cells were carefully washed for 3 × 5 min with PBS and incubated with secondary antibodies for 1 h at RT. Cells were washed as described above, with NucBlue™ Fixed Cell ReadyProbes™ Reagent (DAPI) included in the second set and imaged in PBS. Confocal images were acquired on a Zeiss LSM 880 microscope (Carl Zeiss AG, Oberkochen, Germany) with a 20× or 63× objective. Quantification of the TH-positive fraction was done with a custom-made program in Image-Pro AI for Life Sciences v11.2 (Media Cybernet, RRID SCR_016497).

### 4.9. Proteomics

The pipeline for proteomics analysis is previously detailed in [28]. Cells were washed three times in pre-chilled PBS and a lysis buffer (50 mM Tris-HCI, 50 mM NaCl, 1% SDS, 1% Triton X-100, 1%NP-40, 1% Tween 20, 1% glycerol, 1% sodium deoxycholate (wt/vol), 5 mM EDTA) supplemented with 5 mM dithiothreitol (DTT), benzonase and protease inhibitor, which were added just before collection. Samples were heated to 65 °C at 1200 rpm for 30 min prior to the addition of iodoacetamide IAA (10 mM) and stored at −80 °C until analysis. Samples were run with six technical replicates.

## 5. Conclusions

In summary, we show that coupling geneticin resistance to endogenous TH expression can be used to standardize dopaminergic cultures from iPSC lines derived from different individuals. This improved consistency in dopaminergic neuronal cultures brought by this approach is particularly useful when evaluating rare clinical samples to identify disease-relevant phenotypes.

## Figures and Tables

**Table 1:** Summary of DA neuron enrichment strategies.

Method	Strength	Weakness
Immunolabeling	FACS possible Used for clinical purposes	Require dissociation of cells [21]
TH-knock in fluorescent reporter	Visualization in live cells FACS sorting possible	Require dissociation of cells Genomic editing [32]
NGN2-based	Efficient differentiation in combination with commercial kit Fast differentiation	Genomic editing [17]
Mitomycin C selection	No genomic editing No dissociation	Not specific to DA neurons [19]
MAP2-*neo*	No dissociation	Not specific to DA neurons [26]

## Data Availability

The data, code, protocols, and key lab materials used and generated in this study are listed in a Key Resource Table alongside their persistent identifiers at 10.5281/zenodo.18876867. An earlier version of this manuscript was posted to bioRxiv on 28 February 2025 at https://doi.org/10.1101/2024.02.25.581985.

## Supplementary Materials

The following supporting information can be downloaded at https://www.mdpi.com/article/10.3390/ijms27219394/s1.
