# Supplementary material for: A New Selection Strategy to Enrich Dopaminergic Neurons Facilitates Comparisons Between Patient iPSC-Derived Lines
Source: Int J Mol Sci. 2026 Oct 22;27(21):9394. doi: 10.3390/ijms27219394 (PMC13763022; doi:10.3390/ijms27219394)

Supplementary Table S1. Demographic characteristics of study participants.

Patient / iPSC	*GBA1* genotype	Gender	GD dx age	PD dx age	Biopsy age	RRID (original/TH-neo)
**HT707**	N370S/ c.203del+R257X	F	12	N/A	60	CVCL_F0Y7/ CVCL_F0Y8
**HT708**	N370S/ c.203del+R257X	F	8	55	56	CVCL_F0Y9/ CVCL_F0YA
**HT711**	WT/WT	F	N/A	N/A	58	CVCL_F0YB/ CVCL_F0YC

Further details can be found in Hertz et al. *Evaluation of Induced Pluripotent Stem Cell-Derived Dopaminergic Neurons from Siblings with Gaucher Disease Discordant for Parkinsonim.* Mov Disord. 2025 Aug;40(8):1719-1724. doi: 10.1002/mds.30273.

Supplementary Table S2. Off target predictions for gene editing

gRNA 1

**Sequence**	**PAM**	**Score**	**#MM**	**Gene**	**Locus**
GACGCCGTGCACCTAGCCAA	TGG	N/A		TH	chr11:+2164220
GAAGACATGCACCTAACCAA	AGG	20	4		chr9:+122781796
TACCCCTTGTACCTAGCCAA	TGG	22	4		chr11:+103385851
TATGCCTTGCACCTAGCCTA	GGG	27	4		chrX:+8294520
GACAGCGTGCACCAAGCCAA	GGG	42	3		chr14:-105497759
GACACCGTTCA-CTAGCCAA	TGG	46	3		chr20:+36216921
GACACTGTGCACCTGGCCAA	GGG	46	3		chr10:+123938605
GAGCCCCTGCACCTGGCCAA	GGG	48	4		chr9:-99257132
GAAGCCAGGCACCCAGCCAA	AGG	48	4		chr12:-57556744
GCCGCCGCGCACCTGGCCCA	GGG	49	4	TBX3	chr12:-114674450

gRNA 2

Sequence	PAM	Score	#MM	Gene	Locus
CTAGGTGCACGGCGTCCCTG	AGG	N/A		TH	chr11:-2164216
CAAGGTGAACAGAGTCCCTG	GGG	38	4		chr7:-14579883
CAAGGTTCATGGCATCCCTG	GGG	48	4		chr10:+102459776
CTGGGTAGACGGGGTCCCTG	AGG	50	4		chr20:-23201207
CAAGGTGCCCCCCGTCCCTG	TGG	51	4		chr2:-120738111
CTGGATGCACAGCCTCCCTG	AGG	52	4	C11orf44	chr11:+130716532
CTAGGTACATGGC-TCCCTG	AGG	58	3		chr2:-4064805
CTAGATTCCCGGCATCCCTG	TGG	59	4		chr5:+133918693
CTGGGCGCACGGTGACCCTG	CGG	63	4	ADCK2	chr7:+140672971
CCAGGTGCTCGGCG-CCCTG	CGG	65	3	RMDN3	chr15:+40755272
CTAGAGTGCATGGCTTCCCTG	TGG	65	3		chr11:+80047044

Supplementary Table S3. Primers used and cycle conditions.

Set	
1 Forward	5'-AGCCCTCTAGCCTCATCCTC
1 Reverse	5'-ACATCCCCTGCTTGTTTCAACAG
2 Forward	5'-CCCTCAGACCCTTTTAGTCAGTGTG
2 Reverse	5'-CCAGCCAGCACAGTTTGTGAC

Pair 1 – 40 cycles

Temperature (°C)	Time
95	10 min
95	30 s
57	30 s
72	1 min
72	7 min

Pair 2 – 40 cycles

Temperature (°C)	Time
95	10 min
95	15 s
61	30 s
72	1 min
72	7 min

Supplementary Table S4. Antibodies used in this study

**Antibody**	**Company**	**Catalogue number /RRID**	**Application**
GAPDH	abcam	Ab9485 / AB_307275	WB (1:2000)
TH	Pel-Freeze	P60101-150 / AB_461070	IF (1:400)
TH	Pel-Freeze	P40101-150 / AB_2313713	FC (1:400), WB (1:50)
FoxA2	Neuromics	RA34008 /AB_3719398	IF (1:500)
Neo II	Sigma	06-747 / AB_310234	IF (1:500)

Supplementary Table S5. Summary of materials

Material	Vendor	Catalog number
Fetal bovine serum (FBS)	R&D	G22133,
penicillin-streptomycin	Thermo Fisher	15140-122
CytoTune™-iPS 2.0 Reprogramming System	Thermo Fisher	A16517
Essential 8 Medium	Thermo Fisher	A1517001
Vitronectin	Thermo Fisher	A14700
Y-27632	R&D	1254
StemPro-Accutase	Gibco	A1110501

Lipofectamine Stem Transfection Reagent	Invitrogen	STEM00001
Stemflex	Thermo Scientific	A3349401
Puromycin	Thermo Scientific	A11138-03
Emricasan	R&D	7310
ISRIB	R&D	5284
Polyamines	Sigma	P8483
QuickExtract	Lucigen	QE09050
AmpliTaq Gold 360 Master Mix	Thermo Scientific	4398881
Colcemid	Roche	10295892001
Geltrex	Gibco	A1413302
Neurobasal Medium	Thermo Scientific	21103-049
GlutaMAX	Gibco	35050079
N2	Thermo Scientific	17502048
B-27 without Vitamin A	Thermo Scientific	12587010
Sonic Hedgehog/Shh (C24II) N-Terminus	R&D	1845-SH-500
LDN 193189 dihydrochloride	R&D	6053
A 83-01	R&D	2939
CHIR99021	R&D	4423
SAG 21k	R&D	5282
BDNF	Peprotech	450-02
GDNF	Peprotech	450-10
ascorbic acid	Sigma- Aldrich	4034
TGFβ3	R&D	243-B3
dibutyryl cAMP	Sigma- Aldrich	D0627
DAPT	R&D	2634
Geneticin	Gibco	10131-035,
5-Fluoro-2'-deoxyuridine	Sigma- Aldrich	F0503
Uridine	Sigma- Aldrich	U3003
CellTiter-Glo	Promega	G7570
EDTA-free protease inhibitor	Pierce	A32965
RIPA	Millipore	20-188
BCA	Thermo Scientific	23225
Jess	Bio-Techne	004-650
EZ Standard Pack 1	Bio-Techne	PS-ST01EZ-8
Fluorescence Separation Capillary Cartridges	Bio-Techne	SM-FL001
RePlex	Bio-Techne	RP-001

PBS	Thermo Scientific	14190144
Membrite	Biotium	30093
Papain	Worthington	LK003176
DNAse	Worthington	LS006333
DMEM/Glutamax	Thermo Scientific	10566-016
paraformaldehyde	Santa Cruz Biotechnology	sc-281692
normal donkey serum	Jackson ImmunoResearch	AB_2337258
Isotype control	R&D	AB-105-C
Conjugated secondary antibodies	Jackson ImmunoResearch	711-605-152<br>711-545-152
NucBlue Fixed Cell ReadyProbes Reagent	Thermo Scientific	R37606
8-well imaging chambers	Ibidi	80807-90
NucBlue™ Fixed Cell ReadyProbes™ Reagent	Thermo Scientific	R37606
IAA	Thermo Scientific	A39271

Supplementary Figure S1: Original Western blots

Figure S1 original WB

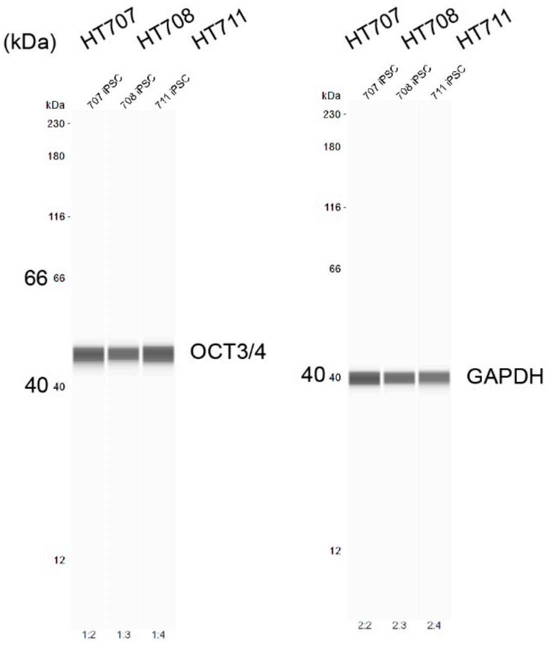

Figure 3 original WB

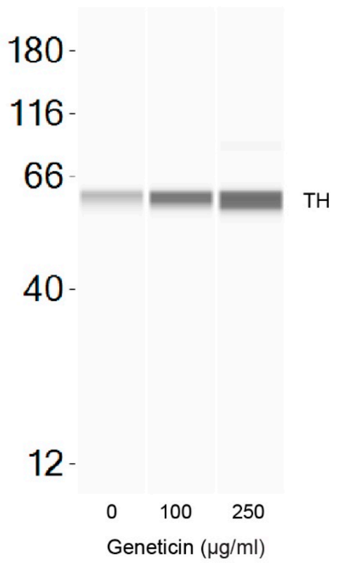

Supplement: Supplementary file 1 [file ijms-27-09394-s001.zip › ijms-4373888-supplementary.pdf]
